# How does capability reconfiguration impact the innovation performance of Chinese manufacturing firms?

**DOI:** 10.3389/fpsyg.2022.966653

**Published:** 2022-07-28

**Authors:** Pan Hu, Yanzhi Hao, Gangyi Wang

**Affiliations:** ^1^College of Economics and Management, Northeast Agricultural University, Harbin, China; ^2^Postdoctoral Research Station of Heilongjiang Property Rights Trading Group, Harbin, China

**Keywords:** innovation magnitude, catch-up in China, capability substitution, capability evolution, incremental innovation, radical innovation

## Abstract

This study explores the relationship between capability reconfiguration and firm innovation performance by analyzing a sample of 375 manufacturing firms in China. The results suggest that the relationship between capability reconfiguration and innovation performance is affected by both the catch-up stage and the mode of capability reconfiguration (evolution or substitution). The catch-up stage of enterprises significantly impacts the moderating effects of innovation magnitude on the relationship between capability substitution and firm innovation performance, however, it has no obvious effects on the moderation of innovation magnitude on the relationship between capability evolution and innovation performance. This study contributes to the theory of dynamic capability and catch-up by revealing how innovation magnitude affects capability reconfiguration and subsequent innovation performance in different catch-up stages. The implication of this study is to remind managers to take full account of the innovation magnitude and catch-up stage in their decision-making.

## Introduction

It is now widely recognized that innovation plays an important role in enhancing an enterprise’s competitive advantage (Davis and Tomoda, 2018; Udriyah et al., 2019; Distanont and Khongmalai, 2020; Yang and Wu, 2021). A firm’s innovation capability mainly lies in its capability both to integrate and build upon its current resources and competencies, while simultaneously developing fundamentally new capabilities, particularly within the late-industrial context (Bogers et al., 2019; Ferreira et al., 2020). Capability reconfiguration, as a key dynamic capability (Lavie, 2006; Ovuakporie et al., 2021), has been considered as an important means to promote enterprise innovation and maintain a competitive advantage in a dynamic environment (Helfat and Peteraf, 2015; Girod and Whittington, 2017; Teece, 2018). Capability reconfiguration occurs when a firm engages in adding, redeploying, recombining, and divesting resources to maintain or enhance competitive advantage in a dynamic environment (Karim and Capron, 2016; Zhou et al., 2019). There are two capability reconfiguration mechanisms: (1) capability evolution, which involves continuous improvement of particular routines; and (2) capability substitution, which offers an immediate and strong response to environmental change (Lavie, 2006).

A great deal of literature on dynamic capability and strategic management of innovation shows that capability reconfiguration has a significant impact on firm innovation (Lavie, 2006; Karim and Capron, 2016). Capability evolution and capability substitution affect corporate innovation in different ways and paths (Girod and Whittington, 2015; Thomas and Douglas, 2022; Xie et al., 2022). Evolutionary capability reconfiguration is the recombination and redeployment of internal and external resources of firms that, helps enterprises discover and capture new opportunities (Wogwu and Hamilton, 2018; Saura et al., 2021). In a rapidly changing environment, the core rigidity and organizational inertia of enterprises will prevent them from making more organizational changes and technological or market-based innovation, thus requiring the necessary changes to suit the rapidly changing technology environment and market environment (Teece, 2007; Bai and Wang, 2016). Through the integration of existing resources and the reconstruction of current capabilities, enterprises can repair, improve some organizational routines, change the old system not suitable for innovation, and update the management strategy adapted to the innovation and competitive environment (Xie et al., 2022). Therefore, from this point of view, capability evolution is usually able to release the potential of the resources leading to innovation (Girod and Whittington, 2017; Ovuakporie et al., 2021). Substitutional capability reconfiguration allows firms to substitute new capabilities for existing capabilities through fundamental change and renewal of organizational capabilities and innovation mechanisms (Lavie, 2006; Hu et al., 2021). Sometimes the local capability adjustment is difficult to completely change the original organizational routine, and the impact of the original convention remains strong. At this time, breaking the original rules and order, to implementing a complete substitution of the overall capability portfolio, is more conducive to the realization of disruptive technological innovation (Lavie, 2006; Karim and Capron, 2016; Girod and Whittington, 2017). So far, the mechanisms by which incremental and rapid innovation impact capability reconfiguration have not been fully revealed (Li et al., 2022; Wang H. et al., 2022).

Corporate decision-makers may need to confront the difficult choices of different capability reconfiguration paths or mechanisms when faced with radical or incremental innovation (Peng et al., 2021; Zhang Z. et al., 2021). The concept of incremental innovation and radical innovation is divided from the perspective of innovation magnitude (Pini and Santangelo, 2010). Incremental innovations are the minimal improvement and minor adjustments to the existing technology (Munson and Pelz, 1979), which involve continuously refining, and exploiting within an existing current technological trajectory (Dewar and Dutton, 1986), while radical innovations represent a risky departure away from an existing technological trajectory (Dosi, 1982; Schoenmakers and Duysters, 2010; Ashford and Hall, 2011). A lot of the literature focuses on the study of the concepts (Dewar and Dutton, 1986; Chandy and Tellis, 1998; McDermott and O’Connor, 2002; Zhang and Chen, 2011), characteristics (Henderson and Clark, 1990; Leifer et al., 2001; O’Connor and Veryzer, 2001; Zhang and Chen, 2011), differences (Danneels, 2004; Fu and Zhang, 2004; Wang H. et al., 2022) and influencing factors (Sandberg and Aarikka-Stenroos, 2014; Simms et al., 2021) of the two modes of innovation. There are also several pieces of literature discussing the possible impact of capability reconfiguration on incremental or radical innovation, such as capability evolution and capability substitution have asymmetric effects on incremental and radical innovation performance (Lennerts et al., 2020; Ovuakporie et al., 2021), and capability evolution and capability substitution generation have quite different effects on enterprise radical innovation in the long and short term (Liu and Su, 2022).

Some researchers find that different innovation magnitudes generate distinct organizational effects on firm capability development, innovation outcomes, and performance (Woschke et al., 2017; Gomes et al., 2019; Tiberius et al., 2021). For example, the strength of radical innovation affects the choice of enterprise capability reconfiguration (Peng et al., 2021). Some empirical studies also show that radical innovation has a positive impact on the substitutional capability reconfiguration, and ultimately it will bring better firm performance (Kim and Mauborgne, 1997; Henard and Szymanski, 2001). However, few studies indicate how the impact of capability evolution and capability substitution on innovation performance varies in different innovation magnitude scenarios.

In the last decade, a growing body of literature on innovation strategic management pays more attention to the dynamic development of firm capabilities, especially the evolution of innovation capabilities of backward enterprises in the process of technology catch-up (Alpkan and Gemici, 2016; Saura et al., 2021). For companies in different stages of catch-up, their technology and knowledge stocks are different, and the capability development path and innovation performance of enterprises should also be different (Hu et al., 2021). However, few studies take the catch-up stage as an important moderator of the impact of innovation magnitude on the relationship between capability reconfiguration and firm innovation performance, although it may become an important variable that influences the direction and way of capability reconfiguration (Kim, 1998; Dutrénit, 2004). For latecomer enterprises, capability reconfiguration is not an overnight change, but a process accompanied by enterprise technology catch-up (Wang, 2018). For example, studies show that latecomer firms promote capability evolution through accumulating knowledge and perpetuating organizational practices in the initial catch-up stage; but they replace old knowledge with new knowledge and reconstruct new organizational practices to achieve capability substitution in the industry frontier stage (Peng et al., 2021). Therefore, different reconfiguration models, innovation magnitude, and catch-up stages will all have a certain impact on the innovation performance of enterprises (Girod and Whittington, 2017; Hu and Yu, 2017; Wang, 2018; Hu et al., 2021).

Therefore, the goal of this paper is to reveal the inherent evolutionary mechanism of capability reconfiguration by examining how innovation magnitude moderates the relationship between capability reconfiguration and firm innovation performance and how the moderating effects vary during different stages of catch-up.

The research questions involved in this study are as follows:

Q1: Whether and how the incremental/rapid innovation affects the relationship between capability and performance?

Q2: Whether and how the impact mentioned above changes as latecomer enterprises are in different catch-up stages?

To answer the above two questions, we seek to achieve the following objectives:

To clarify the three groups of relative concepts: capability evolution/substitution, incremental/rapid innovation, and early/late stage of catch-up.

To establish a measurement of core variables (e.g., catch-up stage).

To explore how the innovation magnitude moderates the relationship between capability reconfiguration and innovation performance, establish a theoretical model and test the size and direction of the moderating effect.

To reveal the path and mechanism of the moderation of innovation magnitude changing with the growth process of the latecomer enterprises, by creating an expansion model containing the variable of catch-up stage, to test and find how the interaction between the catch-up stage and the innovation magnitude affects the relationship between the capability configuration and innovation performance.

The novelty of this paper lies in that, first of all, previous empirical studies have either used incremental/radical innovation as the dependent variable (Li and Qu, 2017; Han et al., 2018, Han et al., 2020; Thneibat, 2021) or as the explanatory variable (Baker et al., 2014; Kim et al., 2019). In present studies, the innovation magnitude is taken as the moderating variable to investigate the path selection and the performance of capability reconfiguration under the background of incremental or/and radical innovation. More importantly, the view of capability evolution over time is also fully considered in our models. Previous studies have rarely introduced catch-up process variables to analyze the capability evolution of latecomer enterprises in different stages, although the catch-up process may exert a significant impact on the capacity accumulation and reconstruction of enterprises (Dutrénit, 2004; Figueiredo, 2017). The studies build a three-way interaction model of the capability reconfiguration, innovation magnitude, and catch-up stages, which method used by Lu and Sun (2016); Sun et al. (2018), to analyze the dynamic effects of capability reconfiguration of latecomer enterprises and its innovation outcomes in the process of technology catch-up, and to explore the influence of time heterogeneity and innovation environment on the dynamic capability and performance.

## Theory and hypotheses

### Capability reconfiguration and firm innovation performance

According to the resource-based view modified by dynamic capability theory, market position and resource advantage are no longer sufficient foundations for sustainable competitive advantage. With rapidly changing technology, the capability to reconfigure and upgrade routines and organizational competencies are the keys to maintaining and enhancing sustainable competitive advantage (Hwang et al., 2020). This has been designated as capability reconfiguration and refers to the activities by which firms engage when adding, redeploying, recombining, or divesting resources or business units. Lavie (2006) suggested: “capability reconfiguration mechanisms are distinct from the notion of dynamic capability, the notion of dynamic capability indicates whether the incumbent can alter the configurations of its capabilities, whereas the notion of capability reconfiguration mechanism suggests how these configurations are likely to change.” According to Lavie (2006), the notion of capability reconfiguration is the integration of Raudino (2016)’s views on technological discontinuities with the perspective of dynamic capabilities. The result is that capability evolution and capability substitution may be considered two extremes of the same continuum. Evolution builds on dynamic capabilities and evolutionary economics to offer an evolution mechanism by which existing capabilities can be adapted. Substitution offers a mechanism of discontinuous change resulting from innovation in which newly acquired capabilities replace capabilities that have been rendered obsolete.

Capability reconfiguration is necessary to match the pace of environmental change (Eisenhardt and Martin, 2000). Reconfiguring resources (whether of internally developed or acquired product lines) and using them in different ways or new combinations provides firms with innovative opportunities (Teece, 2007; Helfat and Peteraf, 2015; Zhou et al., 2019; Khan et al., 2020). Theorists often distinguish between two reconfiguration mechanisms: capability evolution and capability substitution (Karim, 2006; Lavie, 2006). Capability evolution involves the continuous improvement of particular routines. In a rapidly changing environment, a firm’s core competencies will become core rigidities which can cause the firm to lose competitive advantage (Teece, 2007). Therefore, the only way for a firm to sustain a competitive advantage is to continuously invest in and update its resources and capabilities (Lu et al., 2015). Integrating continuous evolution within existing organizational principles, capability evolution is necessary for a firm to match the pace of environmental change (Girod and Whittington, 2017).

On the other hand, capability substitution offers an immediate and strong response to environmental change at the level of the overall capability portfolio. Capability substitution involves changes in fundamental principles of organizational capabilities. Although capability substitution involves relatively large capability changes, (i.e., capability updates, renewals, and iterations at the level of the overall capability portfolio) the configuration of existing capabilities tends to remain intact and organizational design and principles can remain invariant (Lavie, 2006). By changing many elements of the capability portfolios at the same time, capability substitution can avoid the asynchrony of organizational routine adjustment (Haapanen et al., 2016). Compared with capability pitching and adjusting, capability destroying and acquiring are more likely to break core rigidities and path dependencies (Lavie, 2006). Therefore,

**Hypothesis 1a:** Capability evolution is positively related to firm innovation performance.

**Hypothesis 1b:** Capability substitution is positively related to firm innovation performance.

### The moderating effects of innovation magnitude on the relationship between capability reconfiguration and firm innovation performance

Different innovation magnitudes may have divergent effects on organizational capability development and performance (Migdadi, 2019). Existing literature suggests that technological innovations can be divided into incremental innovations and radical innovations that reflect the magnitude of technological innovations (Schoenmakers and Duysters, 2010; Lin and Chang, 2015).

Two characteristics distinguish incremental innovations from radical innovations. The first difference between the two innovative models is embodied in the technological trajectory. The technological trajectories of incremental innovations are linear and continuous, while the technological trajectories of radical innovations are divergent and discontinuous. In other words, incremental innovations involve continuous improving, refining, and exploiting existing current technological trajectories (Schoenmakers and Duysters, 2010), while radical innovations represent a risky departure away from existing technological trajectories (Dosi, 1982). Another difference between incremental and radical innovations is the way a firm allocates existing resources and capabilities. Incremental innovations are based on the existing resources and capabilities of the enterprise and involve continuous improvements or minor adjustments in current technology (Schoenmakers and Duysters, 2010). Radical innovations, however, can ruin existing technology and even destroy existing resources and capabilities, which represent fundamental changes in technology and a risky departure away from existing routine and practice (Mohr, 1981). These different characteristics also influence the impact of capability evolution and capability substitution on firm innovation performance. Thus, radical innovations will induce different outcomes than incremental innovations.

Capacity evolution is the gradual adjustment of organizational routines and existing capabilities. The role of capability evolution may be influenced by innovation magnitude. When the innovation magnitude is lower, the enterprise mainly takes the incremental innovation, which is linear and mild. Incremental innovation is often based on existing knowledge and continually improves current technology by reusing, complementing, and extending the present knowledge (Lin and Chang, 2015). Under the context of incremental innovation, the positive effects of capability evolution would play a better role. Capability evolution is to repair and improve existing capabilities at less cost of change. However, capability substitution is often an overall or fundamental change in capabilities which is costly and risky in the context of incremental innovation and may even have a negative impact on innovation outcomes (Girod and Whittington, 2015, 2017).

With the increase in innovation magnitude, enterprises are more and more inclined to radical innovation. Radical innovation is a non-linear and revolutionary technological change, often accompanied by an update of the technological paradigm and a transition of a technological path (Rialti et al., 2019). Therefore, knowledge creation, technology innovation, and capability iteration are very important to the success of innovation. The greater the innovation magnitude, the greater the expansion of the enterprise knowledge set, and the decrease of dependence on existing knowledge. Capability evolution induces firms to partial adjustment of routines and activities (Rialti et al., 2019) and the local pitching may have overall negative knock-on effects (Girod and Whittington, 2017). Relative to capability evolution, capability substitution involves changes in fundamental organizational principles and can provide firms with access to new solutions (Tetlock, 2007) and replacement of existing capability (Lavie, 2006; Girod and Whittington, 2017). Therefore,

**Hypothesis 2a:** Innovation magnitude weakens the positive relationship between capability evolution and firm innovation performance.

**Hypothesis 2b:** Innovation magnitude strengthens the positive relationship between capability substitution and firm innovation performance.

### The re-moderating effects of catch-up stages on the moderation of innovation magnitude on the relationship between capability reconfiguration and firm innovation performance

Beyond considering the impact of innovation magnitude on the relationship between capability reconfiguration and firm innovation performance, we should also examine the influence of the stage of catch-up. The dynamic resource-based view of the firm argues that organizational capabilities evolve, and proposes that capabilities pass through multiple stages of development before their impact begins to decline (Helfat and Peteraf, 2003). Research on firms in the catch-up stage examined the dynamic processes of their capability building (Kim, 1998; Dutrénit, 2004) and showed that firm capabilities may be accumulated and restructured in different directions and at differing rates (Figueiredo, 2002). Bell (2003) indicated that a technological backward firm, before finally gaining a core technology and becoming an international technological leader, has to go through a period of technological learning and upgrading. Firms upgrading technological capability who are at different stages of catching up are likely to demonstrate different effects in innovation performance depending on whether they use capability evolution or capability substitution. Therefore, we expect that at some point between the early and late stages of catch-up there will be a significant change.

Firms lack basic technological capabilities during their start-up phase. They must first master technical know-how quickly and develop zero-order capabilities (Zollo and Winter, 2002) through learning and imitation. By adding, patching, or deleting routines without change to the overall capability portfolio and structure (Eisenhardt and Brown, 1998; Karim, 2006; Girod and Whittington, 2015), capability evolution can help firms to develop routine capabilities, such as technology-using skills, knowledge, and so on. In the early stage of catch-up, firms have a strong path dependence on existing capabilities, so they must develop fluent organizational routines (Eisenhardt and Brown, 1998). They can do so by using more limited but continuous adjustments instead of substitution, to maintain evolutionary fitness (Teece, 2007). In the case of incremental innovations, firms can use historical experiences and current knowledge more, which will help them to absorb new knowledge more effectively (Han et al., 2018). However, as the magnitude of innovation becomes more radical, the contribution of firms’ existing knowledge and experience to innovation begins to decline. Thus, the positive impact of capability remediation and refinement based on historical experience and existing capabilities on innovation diminishes significantly.

The late stage of catch-up has been termed a transition process from being a laggard to a leader (Dutrénit, 2004). Although firms already have a wealth of knowledge and capabilities, the existing knowledge, skills, experiences, routines, and competencies are all necessary for enterprises to gain a competitive advantage. These existing resources and capabilities are the starting point for enterprises to acquire higher capabilities. Thus, exploiting established competencies provides certain and immediate returns for firms at less cost and risk (Dosi, 1988; Audia and Goncalo, 2007; Phelps, 2010). This is especially true for the case of incremental innovation, which mainly uses the existing knowledge to make the partial adjustment to the production process, products, and technology to enhance short-term performance (Lin and Chang, 2015). However, with the increasing innovation magnitude, firms adopt more radical innovation and will experience a risky departure away from existing routine and practice (Deffains-Crapsky and Klein, 2016). When this occurs the positive impact of capability evolution on innovation will be diminished, or may even eventually turn into negative effects (Phelps, 2010; Girod and Whittington, 2015).

It is generally believed that capability substitution can optimize the capability structure by replacing outdated capabilities with new capabilities, and thereby improve the allocation efficiency of innovation resources. However, the effects of capability substitution on firm innovation performance may be moderated by the magnitude of innovation.

When a firm adopts incremental innovations with low innovation magnitude the firm’s technology innovations only involve minor improvements or simple adjustments in current technology (Dewar and Dutton, 1986). Thus, firms in the early stages of catch-up that adopt incremental innovations can achieve product innovation by tracing leading technology and knowledge and following the basic logic of innovation of following, imitating, and catching up. Decision-makers will replace obsolete existing capabilities with new capabilities that have been proven reliable or mature by technology and markets.

When a firm adopts radical innovations with high innovation magnitude, it has to experience a risky departure away from the existing technological trajectory. In this case, the enterprise will find it very difficult to acquire new capabilities from peer firms and will need to turn to independent research and development. Capability substitution, which involves capability updates, renewals, and iterations at the level of the overall capability portfolio, will lead to higher costs in contrast to more gradual capability evolution. Although capability evolution may also incur short-term performance penalties (Lamont et al., 1994), especially in the early stages of catch-up. Nevertheless, the more radical a firm’s innovation, the more difficult it will be to acquire new technology, and the greater will be the risks and costs of innovation.

In the late stage of catch-up, enterprises already have a certain foundation of knowledge and capability, but the enterprise is striving to achieve technological catch-up and leapfrogging. In this stage, devalued capabilities become core rigidities that handicap the firm in its attempt to adapt to the new environment of competition (Leonard-Barton, 1992). This capability trap, owing to the long-run development of organizational inertia, hinders the innovation and the growth of firms. Substitution can change many elements at the same time to break the core of this capability trap and unleash innovation potential (Girod and Whittington, 2017). The more radical the innovations, the more urgent will be the firms’ appeal to break existing routines and capabilities, and this will lead to greater positive effects on innovation. Therefore,

**Hypothesis 3a:** Catch-up stages do not significantly affect the moderating effects of innovation magnitude on the relationship between capability evolution and firm innovation performance. Innovation magnitude weakens the positive relationship between capability evolution and firm innovation performance in both the early and late stages of catch-up.

**Hypothesis 3b:** Catch-up stages significantly affect the moderating effects of innovation magnitude on the relationship between capability substitution and firm innovation performance. In the early stage of catch-up, innovation magnitude weakens the positive relationship between capability substitution and firm innovation performance. In the late stage of catch-up, innovation magnitude strengthens the positive relationship between capability substitution and firm innovation performance.

## Methodology

### Sample and data collection

We sampled 11 different manufacturing industries. To ensure the validity of the survey responses, the questionnaire was distributed to primary administrators who are familiar with the company’s overall situation. A total of 750 questionnaires were distributed and 290 valid questionnaires were returned for a 38.7% response rate. Of the 290 received, 208 (about 72%) were received initially, and 82 were received at a later stage. Characteristics of the firms and informants in the sample are shown in Table 1. The questionnaire items asked about respondents’ tenure and expertise to verify the appropriateness of the respondents as knowledgeable key informants (Kumar et al., 1993). Overall, 81.4% of the participants had been in their current enterprise for over 6 years. Respondents are mainly managers or top management, and this ensured that they were familiar with firm technological innovation. We checked for nonresponse bias by comparing early and late respondents (Armstrong and Overton, 1977). Results of *t*-tests showed that no systematic differences (*p* > 0.05) were found between the early and late respondents. Thus, non-response bias is not likely not to have affected the results.

**TABLE 1:** Sample characteristics distribution of returned questionnaires.

	Sample	Percentage		Sample	Percentage
Firm size			Firm age (years)		
<500	111	38.28%	<5	43	14.83%
501∼2000	97	33.45%	6∼10	88	30.34%
2000∼5000	65	22.41%	11∼15	37	12.76%
>5000	17	5.86%	16∼20	81	27.93%
			>21	41	14.14%
Ownership			Education		
State-owned	55	32.41%	Doctor	37	12.76%
Private	127	43.79%	Master	89	30.69%
Foreign-funded	94	18.97%	Undergraduate	132	45.52%
Other	14	4.83%	Other	32	11.03%
			Tenure of respondent in firm(years)		
Province			≤5	54	18.62%
Liaoning	42	14.48%	6∼10	119	41.03%
Jilin	35	12.07%	11∼15	87	30.00%
Heilongjiang	54	18.62%	≥16	30	10.34%
Beijing	58	20.00%	Position of respondent		
Tianjin	40	13.79%	Member of executive board	88	30.34%
Shanghai	33	11.38%	Head of R and D	137	47.24%
Other	28	9.66%	R and D project leader	45	15.52%
			Other (e.g., key member of technical expert team)	20	6.90%

We checked for common method variance (CMV) using Haman’s single factor test (Podsakoff and Organ, 1986). We made an orthogonal rotation principal components analysis of all items. The results show that the total explanatory power of the factor reached 77.6%. Moreover, the first factor explained only 17.72% of the variance, which was significantly less than 50%. As a result, the common method variance was unlikely to be a pervasive problem in this study.

### Measures

Survey items were derived from the existing mature scales at home and abroad and were supplemented through field interviews to improve measurement.

Firm innovation performance was measured using a scale adapted from Zhang and Li (2010) and Chen et al. (2011). Firm innovation performance was measured with the following items: (1) novelty of new products, (2) number of new products, (3) speed of new product development, (4) ratio of sales revenue of new products to total sales, (5) new product’s added value and profit margin, and (6) market share of new products. Respondents were asked to give a subjective evaluation of innovation performance from the past 3 years.

Capability reconfiguration was measured using a scale adapted from Gatignon et al. (2002). According to Lavie (2006)’s explanation of capability evolution and substitution, capability evolution means the adjustment and improvement of existing capabilities, and capability substitution includes abandonment of outdated capabilities and acquisition of new capabilities. Thus, our capability evolution measured included 4 items measuring competence-enhancing in the original scale: (1) adjust existing capabilities and practices, (2) develop the existing knowledge base, (3) learn from the existing knowledge, (4) seek solutions from previous experience. In addition, we obtained six items measuring capability substitution by merging new competence acquisition scale items and competence destroying scale items adapted from the original scale: (1) develop new concepts or principles; (2) develop new skills that were not previously available; (3) create new knowledge to replace outdated knowledge; (4) learn knowledge from different knowledge bases; (5) adopt different methods, practices, or processes; and (6) discard obsolete capabilities.

Catch-up stages were measured by two indexes: the technological level and the technological capability of enterprises. According to the existing literature research, the firm catch-up process involves four common stages: starting, following, synchronizing, and leading (Cirera et al., 2020; Peng and Liu, 2021). We ask the interviewees to evaluate the gap in technological levels between their enterprises and the leaders in the past 3 years, and choose their stage in the following options: (1) The gap between us and the leader is huge, and our technology is just beginning; (2) we have a certain gap with the leading enterprise, but we are catching up at full speed; (3) There is no gap between our technology and that of the leading enterprises, which is roughly equivalent; (4) our technology is in the leading position at present, some core technologies are slightly higher than other advanced enterprises. Considering that these measurements may contain some subjective elements that affect the results of the study, we further adopt some mature practices in the existing literature to measure the catch-up stage according to the development of firm technological capability (Xiao et al., 2013; Guo et al., 2015; Park, 2017). The respondents are asked to answer the current state of the enterprise’s technological capability, and there were four items: (1) we are copying the technology of other advanced enterprises or are looking for replicable target enterprises; (2) we are digesting and absorbing the technology of advanced enterprises, and we have also made some initial innovation based on imitation; (3) we have equal R&D cooperation with other leading peers, or we mainly focus on our technology patents and integrate other technologies; (4) we have the capability to innovative technologies and have independent intellectual property rights. According to the answers, we mark each item 1–4 and calculate the average score of the two items, and then judge which stage the firm is in (indicated by the letter D):1 ≤ D < 2 as the initial stage; 2 ≤ D < 3 as the following stage; 3 ≤ D < 4 as the synchronization stage; *D* = 4 as the leading stage. Finally, the initial and the following stages were classified as the early stage of catch-up while the synchronization and the leading stage belonged to the late stage of catch-up.

Innovation magnitude was measured using a scale adapted from Gatignon et al. (2002). Innovation magnitude was measured on a 7-point scale indicating whether each innovation: (1) is a minor improvement over the previous technology (Reversed), (2) is a breakthrough innovation, (3) leads to products that are difficult to replace with substitutes using older technology, and (4) represents a major technological advance in the subsystem. The higher the score was, the more radical the innovation, and the lower the score the more incremental the innovation.

Control variables. Firm size and firm age affect innovation variables such as investment (Baysinger and Hoskisson, 1989; Hoskisson et al., 2002). The larger the firm size, the longer the firm age, the greater the absolute number of resource accumulation, and the more the number of innovative resources (Lee et al., 2001). Therefore, this paper chooses the firm size and firm age as control variables.

### Reliability and validity

Confirmatory factor analysis of variables was carried out using SPSS 21.0 and AMOS 21.0 software. The Cronbach’s alpha of all constructs exceeded 0.7 (Table 2), indicating sufficient reliability for each variable. According to Hair et al. (2013), we deleted the items “discard obsolete capabilities” which resulted in the Chi-square Freedom Ratio exceeding 3 and RMSEA exceeding 0.08, indicating sufficient goodness of fit for the model (Zhang and Li, 2010).

**TABLE 2:** Measurement scales.

	Factor loading	CR	AVE	Cronbach’s α
Capability evolution		0.932	0.773	0.932
Adjust existing capabilities and practices	0.853			
Develop the existing knowledge base	0.875			
Learn from the existing knowledge	0.894			
Seek solutions from previous experience	0.894			
capability substitution		0.886	0.598	0.886
Develop new concepts or principles	0.812			
Develop new skills that were not previously available	0.863			
Create new knowledge to replace outdated knowledge	0.851			
Learn knowledge from different knowledge bases	0.791			
Adopt different methods, practices, or processes	0.795			
Innovation magnitude		0.905	0.704	0.904
Innovation is a minor improvement over the previous technology (Reversed)	0.818			
Innovation is a breakthrough innovation	0.824			
Innovation leads to products that are difficult to be replaced with substitute using older technology	0.886			
Innovation represents a major technological advance in subsystem.	0.826			
Innovation performance		0.942	0.701	0.939
Novelty of the new products	0.843			
Number of new products	0.914			
Speed of new product development	0.861			
ratio of new products sales to total sales	0.898			
Innovative profit margins for new products	0.842			
Market share of new products	0.836			

The convergent validity test showed that the SMC value is greater than 0.5, the standard factor loading was greater than 0.7, the composite reliability (CR) value was greater than 0.7, and the average variance of extraction (AVE) was greater than 0.5, demonstrating that the items have good convergent validity (Hair et al., 2013).

We tested the discriminant validity of the model by using the AVE method (see Table 3). The results showed that the square root values of the average variance extracted for each variable were greater than the Pearson correlation coefficient, which indicated that the questionnaire had good discriminant validity (Fornell and Larcker, 1981).

**TABLE 3:** Descriptive statistics and correlation coefficients.

Variables	Mean	SD	Correlation			
			1	2	3	4
Capability evolution	3.66	1.58	**0.88**			
Capability substitution	3.89	1.38	0.38*	**0.77**		
Innovation magnitude	4.01	1.54	0.11*	−0.23*	**0.91**	
Innovation performance	3.95	1.49	0.65**	0.51**	−0.05**	**0.84**

*p < 0.05, **p < 0.01; The bold number in the diagonal position is the square root of AVE, and the others are the Pearson correlation coefficients.

## Analyses and results

We first examined several commonly used indicators of fit: Chi-square degrees of freedom (*x*^2^/*df*), Goodness-of-fit index (GFI), Adjusted goodness of fit index (AGFI), Root-mean-square error of approximation (RMSEA), and Standard root-mean-square residual (SRMR) which tested the absolute fitness (AFI); Normed fit index (NFI) and Comparative fit index (CFI), which represented the incremental fitness indices; Parsimonious normed fit index (PNFI), Parsimonious comparative fit index (CFI), which are simplified fitness indices (Hair et al., 2013). Results showed AFI *x*^2^/*df* 1.499 < 2.00, GFI = 0.872 > 0.85; AGFI = 0.872 > 0.85, RMESE = 0.042 < 0.05, SRMR = 0.049 < 0.05; the incremental fitness index, NFI = 0.936 > 0.90, CFI = 0.978 > 0.95, IFI = 0.978 > 0.95, RFI = 0.927 > 0.90, TLI = 0.975 > 0.95; and the simplified fitness index, PNFI = 0.824 > 0.50, PGFI = 0.732 > 0.50, PCFI = 0.861 > 0.50. These results demonstrated acceptable model fit.

Table 3 shows the descriptive statistics and the correlation matrix of the main variables in our study. We checked the variance inflation factors (VIFs) to investigate the multicollinearity problem. The individual VIFs ranged from 1.005 to 2.293. Given that all the VIFs were far below the commonly accepted value of 10 (Cohen et al., 1983), multicollinearity was unlikely to be a big problem in our study.

Table 4 contains the results from the hierarchical OLS regression analysis. The control variables (firm age and size) were entered in model 1, which indicated that only firm size is found to have a significant effect (*p* < 0.001). The main predictors (capability evolution, capability substitution) were in model 2, the interactions between capability reconfiguration and innovation magnitude were in model 3, and the three-way interactions between capability reconfiguration, innovation magnitude, and catch-up stages were entered in model 4. The four regression equations were significant at *p* < 0.05, and the adjusted R2 values range from 0.115 for model 1 to 0.778 for model 4. In addition, we mean-centered the interactions to reduce multicollinearity. All values of the resulting variance inflation factor were lower than 2.0, which indicated that multicollinearity was not a concern.

**TABLE 4:** Results of hierarchical linear regression analysis for firm innovation performance.

Dependent variable: Firm innovation performance	Model 1	Model 2	Model 3	Model 4
**Control variables**				
Firm age	0.011(0.012)	0.010(0.009)	0.012(0.008)	0.005(0.006)
Firm size	0.287(0.047)***	0.157(0.037)***	0.127(0.032) ***	0.090(0.025)***
Independent variables				
Capability evolution		0.462(0.043)***	0.889(0.071)	0.519 (0.113)***
Capability substitution		0.295 (0.049)***	0.285(0.052)	0.478(0.195)*
Innovation magnitude			0.138(0.111)	−0.076(0.115)
Catch-up stages				−0.127(0.146)
**Interaction between variables**				
Capability evolution × Innovation magnitude			−0.301(0.074)***	−0.352(0.130)**
Capability substitution × Innovation magnitude			0.902(0.088)***	−1.202(0.210)***
Innovation magnitude × Catch-up stages				0.467(0.186)*
Capability evolution × Catch-up stages				−0.082(0.189)
Capability substitution × Catch-up stages				0.582(0.231)*
Capability evolution × Innovation magnitude × Catch-up stages				−1.013(0.272)***
Capability substitution × Innovation magnitude × Catch-up stages				1.595(0.291)***
Adjusted R^2^	0.109	0.484	0.621	0.767
ΔR^2^	0.115	0.376	0.139	0.148
ΔF	18.728***	105.183***	35.315***	30.524***

Standard errors in parentheses: *p < 0.1, **p < 0.05, ***p < 0.01.

Hypothesis 1 predicted that both capability evolution and capability substitution are positively related to firm innovation performance. The results of our hierarchical linear regression analysis in Model 2 (see Table 4), supported this hypothesis, revealing a significant positive relationship between capability evolution, capability substitution, and firm innovation performance (β_1_ −0.462, *p* < 0.05; β_2_ 0.295, *p* < 0.05).

Hypothesis 2 predicted that innovation magnitude weakens the positive relationship between capability evolution and firm innovation performance while strengthening the positive relationship between capability substitution and firm innovation performance. Results of model 3 presented in Table 4 supported this hypothesis, revealing a significant negative interaction between innovation magnitude and capability evolution (β_1_ = −0.301, *p* < 0.001), and a significant positive interaction between innovation magnitude and capability substitution (β_2_ = 0.902, *p* < 0.001). The results of regression analysis indicated that the more innovation tends to breakthrough, the smaller the positive impact of capability evolution on firm innovation performance, and the greater the positive impact of capability substitution on the firm.

**FIGURE 1:**
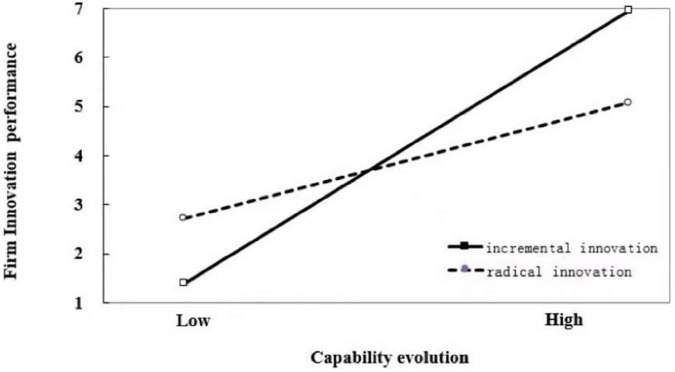
Moderating effect of innovation magnitude on the relationship between capability evolution and firm innovation performance in the early stage of catch-up.

**FIGURE 2:**
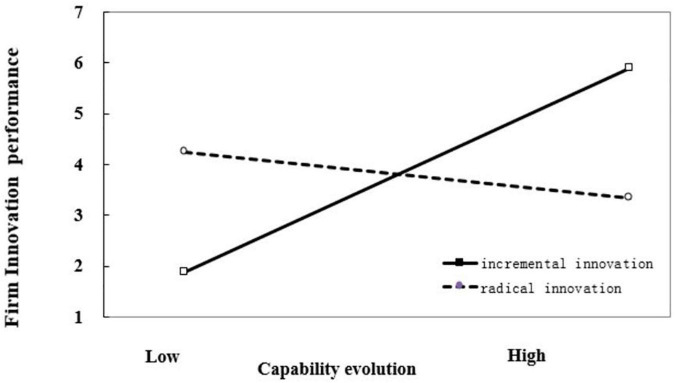
Moderating effect of innovation magnitude on the relationship between capability evolution and firm innovation performance in the late stage of catch-up.

**FIGURE 3:**
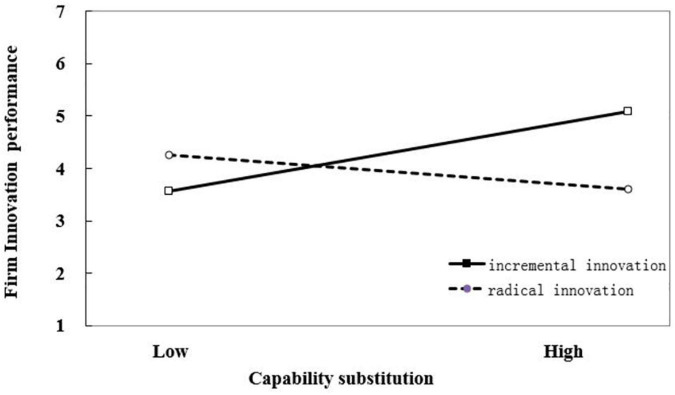
Moderating effect of innovation magnitude on the relationship between capability substitution and firm innovation performance in the early stage of catch-up.

**FIGURE 4:**
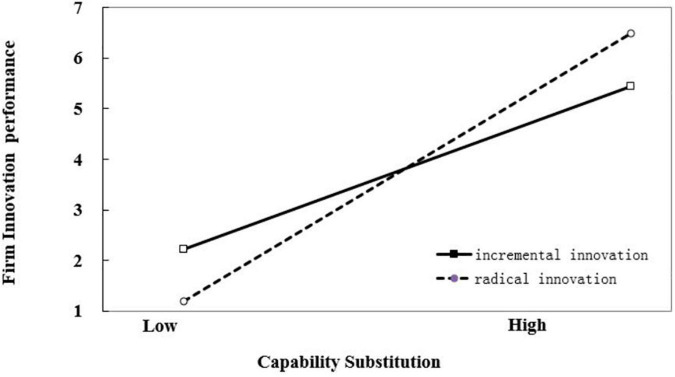
Moderating effect of innovation magnitude on the relationship between capability substitution and firm innovation performance in the late stage of catch-up.

Hypothesis 3 predicted that catch-up stages would not have a significant impact on the interaction between capability evolution and innovation magnitude, while catch-up stages would change the interaction between capability substitution and innovation magnitude. The results of our hierarchical modeling analysis in Model 4, supported this hypothesis. The interaction coefficients between capability evolution and innovation magnitude (β_1_ = −0.352, *p* < 0.01), as well as the three-way interaction coefficients between capability evolution, innovation magnitude, and catch-up stages (β_2_ = −1.031, *p* < 0.001) were all significantly negative, indicating that innovation magnitude weakens the positive relationship between capability evolution and firm innovation performance in both early and late stages of catch-up. The interaction coefficient between capability substitution and innovation magnitude was significant negative (β_1_ = −1.202, *p* < 0.001), while the three-way interaction coefficient between capability evolution, innovation magnitude, and catch-up stages was significantly positive (β_2_ = 1.595, *p* < 0.001), indicating that catch-up stages significantly affected the moderating role of innovation magnitude on the relationship between capability substitution and firm innovation performance. The results showed innovation magnitude weakens the positive relationship between capability substitution and firm innovation performance in the early stage of catch-up while strengthening the positive relationship between capability substitution and firm innovation performance in the late stage of catch-up. The moderating effects of innovation magnitude on the relationship between capability reconfiguration and firm innovation performance in different stages of catch-up are illustrated in Figures 1–4.

## Discussion

In recent years, capability reconfiguration has become an important driving force for enterprises to accelerate innovation and enhance competitiveness (Chen et al., 2022). However, there are still different voices about whether evolutionary and substitutional capability reconfiguration can contribute a positive role to innovation performance (Karim and Capron, 2016; Girod and Whittington, 2017). In our results, both capability evolution and capability substitution are significantly positively correlated with firm innovation performance. This is similar to the results of some other studies (Lavie, 2006; Karim and Capron, 2016; Valdemarin and Mayrhofer, 2022; Xie et al., 2022). Moreover, the path coefficient of capability evolution impacting innovation performance is 0.462 (*p* < 0.001), which is greater than that of capability substitution (β = 0.295, *p* < 0.001). This could mean the evolutionary capability reconfiguration brings more innovation outcomes to firms (Eisenhardt and Brown, 1999; Girod and Whittington, 2015), which is in agreement with the empirical results obtained by Zhang and Lv (2014).

Some studies have also shown that the heterogeneity of innovation magnitude and technical level may become important factors affecting capability reconfiguration and innovation performance (Zhong et al., 2014). In the study, it is found that the interaction coefficient for the two variables, capability evolution and innovation magnitude to innovation performance is −0.301 (*p* < 0.001). It suggests that innovation magnitude weakens this positive effect of capability evolution on firm innovation performance. Specifically, the positive effect of capability evolution on firm innovation performance will be stronger for incremental innovation than for radical innovation. Therefore, it is more suitable for the friendly and mild innovation environment to adjust and perfect the capability from the inside of the enterprise, which is similar to some findings of existing studies (Capron and Mitchell, 2009). When the innovation magnitude is low, the enterprise is mainly committed to the transformation of the technology platform, the improvement of general technical means, and existing products (Hansen and Ockwell, 2014; Figueiredo, 2017), which is exactly the strength of evolutionary capability reconfiguration. Through capability evolution, enterprises update their knowledge and technology, thus deepening their understanding of market knowledge, popular technical means, and current competition. This approach not only achieves better market performance but also reduces the excessive costs of developing new technologies (Shankar et al., 1999). The interaction coefficient of capability evolution and innovation magnitude to innovation performance is 0.902 (*p* < 0.001). It suggests that innovation magnitude strengthens the positive effect of capability substitution on firm innovation performance. In other words, the positive effect of capability substitution on firm innovation performance will be stronger for radical innovation than for incremental innovation. The results show capability renewal based on externally sourced capabilities is more suitable for the innovation environment full of challenge and competition (Capron and Mitchell, 2009). Rapid innovation is the subversion and reconstruction of existing knowledge and technology. Through exploratory learning and capabilities rebuilding, it can get rid of the dependence on the existing knowledge inertia, experience inertia, and learning inertia, which is more conducive to promoting enterprise innovation (Chandy and Tellis, 2000; Li and Zeng, 2019).

Furthermore, our results also show that the impact of innovation magnitude on capability substitution varies at different stages of technological catch-up. This is consistent with previous studies: dynamic capability and innovation strategy of enterprises vary at different stages of technology catch-up (Alpkan and Gemici, 2016; Peng et al., 2021; Zhang L. et al., 2021). The interaction coefficient between capability substitution and innovation magnitude was significant negative (β_1_ = −1.202, *p* < 0.001), while the three-way interaction coefficient between capability evolution, innovation magnitude, and catch-up stages was significantly positive (β_2_ = 1.595, *p* < 0.001). This suggests that the catch-up stage exerts a significant effect on the moderating effect of the innovation magnitude on the relationship between capability substitution and firm innovation performance. In the early stage of catch-up, enterprises in emerging economies had low technology level and weak knowledge reserves (Dutrénit, 2004; Zhong et al., 2014), and innovation based on learning and imitation was more suitable (Mathews, 2002). It is necessary for enterprises to complete their knowledge reserve and gradually complete their capability accumulation, which is the focus of enterprise strategic development (Figueiredo, 2017). Due to the constraints of their organizational inertia and path dependence, the greater the innovation magnitude is adopted, the more aggressive capability reconfiguration will make enterprises face higher innovation costs and a greater risk of failure (Zhong et al., 2014). With the latecomer firms approaching the technological frontier, the technical level and innovation capability have been comprehensively improved (Lv and Su, 2009; Figueiredo, 2017). At this time, if enterprises want to further innovate, they must constantly break the original conventions and practices, and break through the existing knowledge domain and technology set (Hu et al., 2021). Radical innovation has the characteristics of innovating existing technologies, leading the market, and reshaping consumer preferences (Zhou, 2006), which is conducive to enterprises to fundamentally establish the status of “sheep”, and increase their market competitiveness by increasing brand loyalty and other ways (Zhong et al., 2014). And it can further reduce the cost of production and advertising, thus in this stage, the more radical the innovation is, the more conducive to the improvement of enterprise innovation performance.

On the other hand, the negative moderation of innovation magnitude on the relationship between capability evolution and firm innovation performance does not show a directional change in the early and late stages of catch-up. Innovation magnitude always negatively moderates the positive relationship between capability evolution and firm innovation performance both in the whole stages of catch-up. The empirical results further show that the negatively moderating effect of innovation magnitude on the relationship between capability evolution and enterprise innovation performance in the later stage of catch-up is greater than that of the early stage, with the three-way interaction coefficient being negative 1.013 (*p* < 0.001). Xie et al. (2022) noted that capability evolution allows enterprises to adjust the method of value innovation and the direction of product innovation. This capability is undoubtedly to realize the transfer and application of structural knowledge from a familiar domain to a completely new domain (Berends et al., 2016). Capability evolution is much more dependent on the existing organizational routine, knowledge, and experiences (Dang et al., 2013). The lower the magnitude of innovation, the more effective the knowledge inertia can reduce the complexity and uncertainty in innovation, and the more effective the capability evolution based on exploiting learning (Xie et al., 2016; Li and Zeng, 2019). On the contrary, the more radical the innovation is, the more enterprises need to subvert the original technical methods and management philosophy and try more exploratory learning to acquire new knowledge, methods, and skills (Chandy and Tellis, 2000; Liu et al., 2017). In this case, the more difficult the role of capability evolution is to play. Moreover, the closer the enterprises are to the technology frontier, the stronger the demand for the acquisition of new knowledge, methods, and technologies is, and the capability to adjust, add and improve organizational routine will be continuously weakened.

## Conclusion

This study provides a detailed understanding of the mode of capability reconfiguration and innovation magnitude and their important contributions to firm innovation performance. Based on the perspective of dynamic capability, this study uses moderating effect model with three-way interaction variables to examine the impact of innovation magnitude and catch-up stage on the relationship between capability substitution and firm innovation performance. The main conclusions are as follows: Firstly, both capability evolution and capability substitution, as two common forms of capability reconfiguration, have a positive impact on firm innovation performance, which is consistent with the mainstream research findings (Lavie, 2006; Karim and Capron, 2016). Secondly, innovation magnitude was a moderator between capability reconfiguration and firm innovation performance. Innovation magnitude weakens the positive relationship between capability evolution and firm innovation performance, but it strengthens the positive relationship between capability substitution and firm innovation performance. This result shows that the heterogeneity of innovation magnitude ultimately affects the reconfiguration mode of enterprise capability and its effect through the differences in knowledge composition, organizational learning, technological trajectory, innovation strategy, and so on (Li and Zeng, 2019). Thirdly, in the early and late stages of catch-up, there is a great difference in the intensity and direction of the moderation of innovation magnitude on the relationship between capability reconfiguration and innovation performance. The results make a basic conclusion: in the early stage of catch-up, the lower the innovation magnitude, the more obvious the positive role of capability evolution; but in the late stage, the higher the innovation magnitude, the more significant the positive role of capability substitution is. This study contributes to the dynamic capability theory and the catch-up theory: it specifically demonstrates how the dynamic capability reconfiguration path is affected by the technology catch-up process; it also explains that the technology catch-up strategy of the latecomer enterprises should be appropriately adjusted according to the innovation magnitude of the enterprises and the industry. This study contributes to the theory of dynamic capability and catch-up by revealing how innovation magnitude affects capability reconfiguration and subsequent innovation performance in different catch-up stages. It also reminds managers to take full account of the innovation magnitude and catch-up stage in their decision-making.

## Theoretical implications

This study makes several contributions. First, this study contributes to the literature on dynamic capability by providing one of the few empirical tests of capability reconfiguration on firm innovation performance. Through testing and comparing the performance outcomes of two forms of reconfiguration, this research supports the assertion that evolution and substitution have different effects on organizational innovation (Lavie, 2006; Girod and Whittington, 2017). In addition, we further found distinct contributions of capability evolution and capability substitution on innovation outcomes in different catch-up stages. These findings are not only a response to the literature based on the strategic evolution and capability evolution of the enterprises in developed countries (Helfat and Peteraf, 2003; Davis and Tomoda, 2018), but also to explore and describe the capability development path of the latecomer enterprises, which improve and supplement the theory of capability accumulation and capability construction of catch-up enterprises (Dutrénit, 2004; Figueiredo, 2017).

Second, this study contributes to the theory of incremental and radical innovation in several ways. For one thing, departing from past empirical studies which either consider incremental/radical innovation as an explanatory variable (Baker et al., 2014; Kim et al., 2019; Wang et al., 2020) or consider it as being explained (Dunlap-Hinkler et al., 2010; Zhou and Li, 2012; Thneibat, 2021). By using innovation magnitude as a moderator, the present study examines how incremental and radical innovation affects the innovation process and outcome based on a dynamic capability perspective. The finding enriches our understanding of the underlying mechanisms for which innovation magnitudes influence the capability evolution process (Zhang and Lv, 2014; Zhou et al., 2019).

For another, previous studies suggested that firms have distinctly different performances in incremental and radical innovations (Morone, 1993; Woschke et al., 2017), our results not only support this view but also reveal possible mechanisms by which these differences arise. The path dependence, organizational inertia, and the correlation of product innovation on the old knowledge and experience under the background of different innovation magnitude greatly affect the role of capability evolution and capability substitution, which provides new research ideas for rapid innovation and capability reconstruction in the future (Chen and Qiu, 2022; Wang W. et al., 2022).

Third, our study contributes to catch-up theory by verifying the significant effects of catch-up stages on the moderating effects of innovation magnitude on the relationship between capability reconfiguration and firm innovation performance. The existing literature on technological catch-up holds that the catch-up stages of firms may be important variables that influence the direction and magnitude of capability building (Kim, 1998; Dutrénit, 2004). Our research provides an attempt to reveal that the early stage and late-stage catch-up are not the only factors that determine the capability construction of enterprises, the innovation condition (incremental or radical) faced by enterprises is also one of the important factors.

## Practical implications

The findings have implications for managerial practices. Capability reconfiguration is considered an important driver of technology innovation and a firm’s growth (Ovuakporie et al., 2021). Our research suggests that capability substitution is not necessarily the most beneficial way for innovation, and the capability evolution is a model worth considering under relatively moderate innovation magnitude for decision-makers (Zhang and Lv, 2014). In practice, other factors, such as the magnitude of innovation, should be considered in choosing evolution or substitution. If the enterprise is in the traditional manufacturing enterprise, more inclined to gradual innovation environment, capability evolution is more recommended; on the contrary, if the enterprise is high-tech or emerging innovative enterprises, more inclined to radical innovation environment, capability substitution should be the first choice of managers (Zang and Zhang, 2021; Wang D. et al., 2022). More importantly, the strategy makers of the enterprise should clearly understand the development stage and knowledge potential of the enterprise and develop the capability reconfiguration strategy based on fully considering the degree of industrial competition and enterprise innovation magnitude (Liu and Dang, 2013).

## Limitations and further research

First of all, this study focuses on the influence of technological radical/incremental innovations on the dynamic capability and innovation outcomes, which are the most prominent types of innovations in manufacturing firms (Phene et al., 2006; Liu et al., 2020). However, what’s worth noticing is that the other types of innovation such as product innovation and market innovation are also important for a firm’s capability development and performance (Davis and Tomoda, 2018). Future studies can investigate the effects of other types of resource constraints.

Second, we discuss the impacts of innovation magnitude on the relationship between capability reconfiguration and innovation performance in the early and late stages of catch-up, while there are several patterns of catch-up such as path-following, path-skipping, and path-creating (Lee and Ki, 2017), and a few different stages such as initial, following, synchronizing and leading (Sui and Chen, 2015). The development and evolution of the innovation capability of firms may be distinguished in different modes of stages of catch-up (Guo and Zheng, 2019). Future studies can explore the capability evolution and innovation outcome of different modes and stages. Thirdly, a potential extension of this study would be to employ a longitudinal study design to empirically test causality and assess innovation capability and firm performance outcomes over time.

## Data availability statement

The original contributions presented in this study are included in the article/supplementary material, further inquiries can be directed to the corresponding author.

## Ethics statement

Ethical review and approval was not required for the study on human participants in accordance with the local legislation and institutional requirements. Written informed consent from the patients/participants or patients/participants legal guardian/next of kin was not required to participate in this study in accordance with the national legislation and the institutional requirements.

## Author contributions

PH and YH designed the research and methodology, compiled the literature, and put forward the policy recommendations. GW provided the guidance throughout the entire research process. All authors contributed to the article and approved the submitted version.
